# A Perspective on Substorm Dynamics Using 10 Years of Auroral Kilometric Radiation Observations From Wind

**DOI:** 10.1029/2022JA030449

**Published:** 2022-09-12

**Authors:** J. E. Waters, C. M. Jackman, D. K. Whiter, C. Forsyth, A. R. Fogg, L. Lamy, B. Cecconi, X. Bonnin, K. Issautier

**Affiliations:** ^1^ Space Environment Physics Group School of Physics and Astronomy University of Southampton Southampton UK; ^2^ DIAS Dunsink Observatory School of Cosmic Physics Dublin Institute for Advanced Studies Dublin Ireland; ^3^ Department of Space and Climate Physics MSSL UCL Dorking UK; ^4^ Observatoire de Paris LESIA PSL Research University CNRS Sorbonne Université University of Paris Meudon France; ^5^ LAM Pythéas Aix Marseille Université CNRS CNES Marseille France

## Abstract

We study 10 years (1995–2004 inclusive) of auroral kilometric radiation (AKR) radio emission data from the Wind spacecraft to examine the link between AKR and terrestrial substorms. We use substorm lists based on parameters including ground magnetometer signatures and geosynchronous particle injections as a basis for superposed epoch analyses of the AKR data. The results for each list show a similar, clear response of the AKR power around substorm onset. For nearly all event lists, the average response shows that the AKR power begins to increase around 20 min prior to expansion phase onset, as defined by the respective lists. The analysis of the spectral parameters of AKR bursts show that this increase in power is due to an extension of the source region to higher altitudes, which also precedes expansion phase onset by 20 min. Our observations show that the minimum frequency channel that observes AKR at this time, on average, is 60 kHz. AKR visibility is highly sensitive to observing spacecraft location, and the biggest radio response to substorm onset is seen in the 21:00–03:00 hr local time sector.

## Key Points

- Auroral kilometric radiation (AKR) observations made over 10 years are compared with four event lists of substorm onsets using superposed epoch analyses
- On average, AKR power increases and source region extends to higher altitudes in the 20 min prior to onset
- The occurrence of AKR power at higher altitudes is sensitive to the substorm size

## 1 Introduction

Auroral kilometric radiation (AKR) is non‐thermal radio emission generated within a plasma cavity that is extended longitudinally about the terrestrial nightside at high magnetic latitudes (Calvert, 1981; Gurnett, 1974; Mutel et al., 2008; Yearby & Pickett, 2022). Electron populations in the magnetosphere‐ionosphere coupling region, where field‐aligned currents extend from the plasma sheet to the ionosphere, supply the generation of AKR via the electron‐cyclotron maser instability (Wu & Lee, 1979). As such, the emission frequency is very close (typically within 1%–2%) to the electron gyrofrequency, which increases with magnetic field strength as converging field lines reach the auroral zone. AKR has been observed to correlate closely with ionospheric auroral emission, with active source regions existing above brightenings in the auroral oval, typically in premidnight local time (LT) sectors (Huff et al., 1988; Mutel et al., 2004; Panchenko, 2003; Schreiber et al., 2017). As well as auroral brightenings, AKR is also coincident with many of the other observed processes in the magnetosphere that occur during substorms and times of disturbance, such as high velocity flows and geosynchronous particle injections in the magnetotail and increased ground magnetic activity (Fairfield et al., 1999). The activation of a lower frequency AKR source implies that the source region has extended to higher altitudes along the field line, and as such is a proxy for the structure of the auroral acceleration region, which has been confirmed by in situ measurements of the source region (Ergun et al., 1998). The auroral acceleration region is integral to understanding magnetosphere‐ionosphere coupling, and AKR observations have been used to infer its changing morphology during times of disturbance (Morioka et al., 2010).

Substorms are space weather events that are characterized by various plasma dynamics under changing magnetospheric configurations and orientations of the interplanetary magnetic field (IMF). When the dayside reconnection rate is high, often when a southward (negative *B*_*Z*_ in geocentric‐solar‐magnetic (GSM) coordinates) component is present in the IMF, magnetic flux is loaded into the magnetotail as it lengthens and the plasma sheet compresses. This is the growth phase of the substorm (McPherron, 1970). The energy is released into the coupled magnetosphere‐ionosphere as reconnection occurs on the nightside and current is diverted from the magnetotail into the high latitude ionosphere, after which the system either returns to a more stable dipolar configuration (Hones Jr, 1985) in the recovery phase or continues to drive further releases of energy (Akasofu, 2017; Kepko et al., 2015). In practice there is much variability between substorm events, and the exact timeline of contributing processes is not fully understood. Substorm onset, which defines the beginning of the expansion phase, is most often used to align events (e.g., Forsyth et al., 2015; Walach et al., 2017; Wild & Grocott, 2008). Extreme dynamics are seen in the auroral oval as it expands poleward and a bright bulge travels westward in the aurora. Characterization of substorm dynamics, particularly across the growth and expansion phases, was pioneered with the use of networks of auroral all‐sky cameras (Akasofu, 1964) and later with global UV imagers of the oval (Frey, 2004). These extreme auroral changes are coincident with a surge in the westward electrojet (e.g., Weimer et al., 1994), a high latitude current that is driven by the diverted magnetotail current (Forsyth et al., 2014, 2018; Kepko et al., 2015; Lui, 2013; McPherron et al., 1973). The strengthening of this current system is typically used to define onset as it produces a clear signature in the deflection of the Northward component of the terrestrial magnetic field, as measured by ground magnetometer stations. These have historically been combined to produce indices of the activity and continue to do so with good spatial coverage (Newell & Gjerloev, 2011). In situ measurements also allow the phase of the substorm to be inferred, with satellites on the nightside being able to observe dipolarisations in the magnetotail as well as measurements of substorm‐associated electron populations (Juusola et al., 2011; Liou, 2002). This combination of observations has allowed us to determine characteristic times of substorm events, in turn allowing examination of other phenomena during the event timeline (Haiducek et al., 2020).

The aforementioned correlation of AKR with geomagnetic disturbances is particularly highlighted during substorms. This is quantified in studies of the AKR power and the auroral electrojet (AE) index (Kaiser & Alexander, 1977; Voots et al., 1977), field‐aligned currents (Green et al., 1982) and electron precipitation (Imhof et al., 2000). Global observations of the auroral oval at substorm onset have also provided an insight to coincident AKR enhancement (Liou et al., 2000). As well as this, AKR intensifications are typically accompanied by spectral extensions, notably to lower frequencies (Hanasz et al., 2001). These low frequency extensions occur close to substorm onset, and have been studied by the Polar plasma wave instrumentation in conjunction with ground and in situ measurements of the magnetic field, electron populations and other plasma parameters (Morioka et al., 2007, 2010). The spectral changes observed in AKR during these events has allowed, by proxy, the evolution of the auroral acceleration region to be inferred; extending to higher altitudes as source regions of low frequency AKR become active along high latitude magnetic field lines (Morioka et al., 2012). While these studies of AKR have allowed for characterization of this important region of the magnetosphere they are typically conducted over a limited number of events.

We now have an opportunity to significantly extend the study of the link between substorms and AKR due to the availability of years of high fidelity data from the Wind spacecraft. Accounting for viewing limitations, 10 years of calibrated AKR observations from 1995 to 2004 are now able to be examined, with properties of the emission itself and spectral features available (Fogg et al., 2022; Waters, Jackman et al., 2021). This allows coincident lists of substorm events, derived from various observational signatures and that also cover decadal timespans, to be compared with the AKR observations. With the novel data available, we examine the AKR observations during the magnetosphere‐ionosphere coupling timeline of substorms as defined by the aforementioned lists. In this way we aim to characterize the average AKR response with respect to other changes within the magnetosphere, as well as examine how both the intensity and spectral parameters of AKR change with the size of the substorm.

In Section 2 we introduce the AKR data used here, giving the important context of spacecraft viewing to the 10 years of observations, as well as introducing the various lists of substorm events and their associated observational signature. Section 3 details the analysis of the AKR power with the substorm timeline as defined by each of these lists and the interpretation of the results, while Section 4 concerns the analysis and interpretation of the spectral parameters of AKR, providing insight to the typical evolution of the auroral acceleration region. In Section 5 we conclude this work with a summary of the analysis conducted and their primary results.

## 2 Data and Methods

### 2.1 Wind Radio Measurements and AKR Bursts

**Figure 1:**
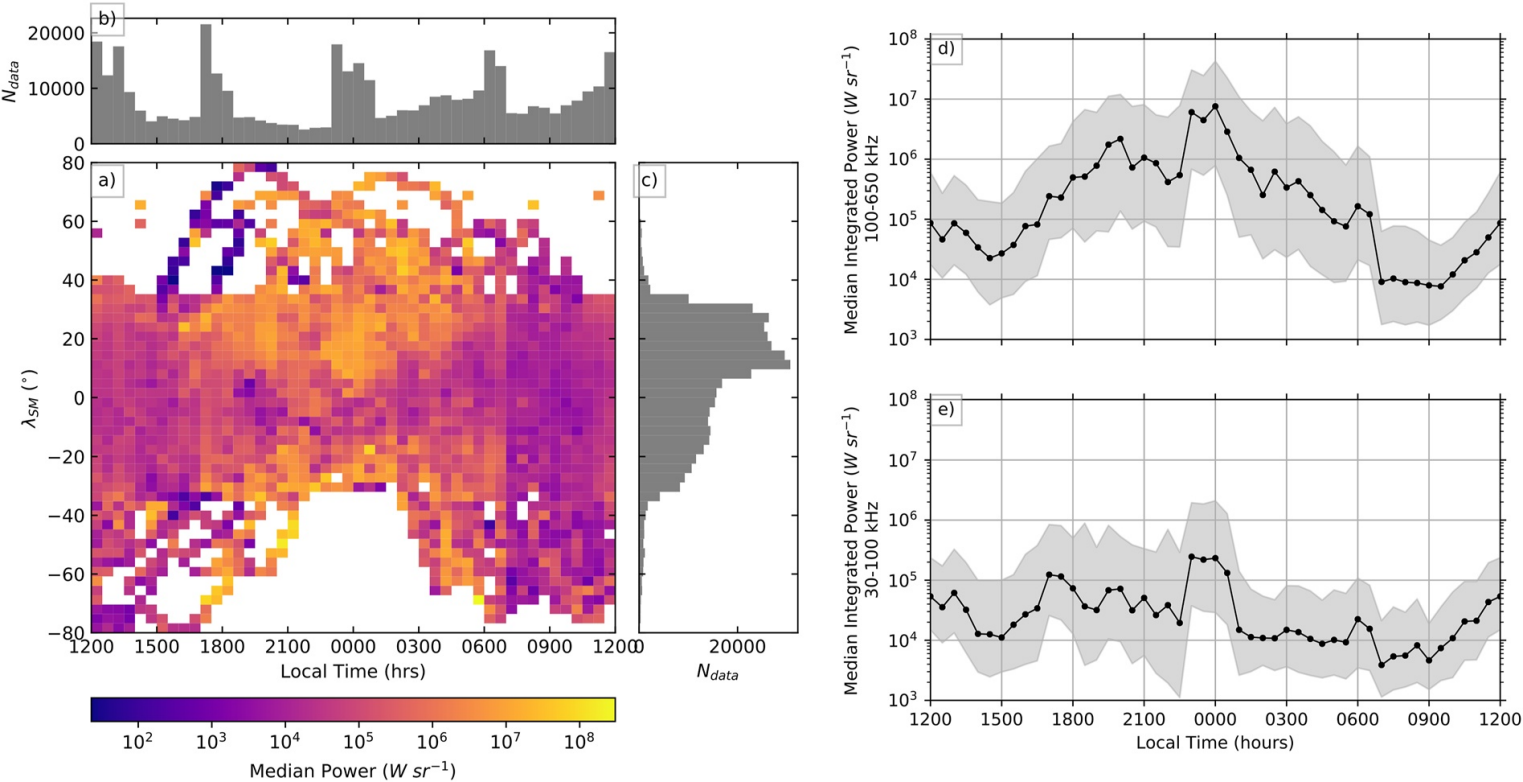
Average auroral kilometric radiation (AKR) power, integrated between 30–650 kHz, (a) for viewing positions of Wind from 1995 to 2004 inclusive, with marginal distributions of spacecraft local time (LT) (b) and magnetic latitude (*λ*_SM_) (c) of the observations shown. Panels (d and e) show the median AKR power for frequency ranges that represent the higher frequency (HF 100–650 kHz) and lower frequency (LF 30–100 kHz) components of AKR, respectively, with the upper and lower quartiles shown.

For this statistical study we use 10 years of radio data from the Wind spacecraft, covering the interval from 1995 to 2004 inclusive. During this time, Wind explored all LT at a range of radial distances and latitudes which allowed it to probe the solar wind and various magnetospheric regions in situ in order to tackle different science objectives (Pelton & Allahdadi, 2015). From mid‐2004 onwards, Wind reached what is to be its final destination, as it entered a Lissajou orbit about the first Lagrangian point L1. For the first 5 years of observations, from ∼1995–2000, Wind observed most often from dayside LTs during precessing orbits with dayside apogees. Near 2000 Wind was sent into a trajectory that took it to radial distances of 250 R_*E*_ (1 *R*_*E*_ = 6,371 km (1 Earth radius)) on the dawn and dusk flanks. From mid‐2003 to mid‐2004, Wind explored the nightside magnetosphere, being placed in a trajectory that sent it downtail to the Lagrangian point L2. The nightside location of the source regions and the highly anisotropic beaming of the emission has consequences for the viewing of AKR for a remote sensing spacecraft such as Wind, and so the spacecraft position at the time of substorm onset must be considered. The RAD1 receiver of the Wind/WAVES instrument takes a variable number of samples (between 1 and 4) of 32 frequency channels, between 20 and 1,040 kHz, over a ∼3 min sweep cycle (Bougeret et al., 1995). Figure 1 shows the median AKR power observed during 1995–2004, binned by the LT and magnetic latitude of Wind during the sweep cycle, as well as the marginal distributions that show the number of sweeps made in each LT and latitude bin respectively. Figure 1a shows the median power integrated over typical AKR frequencies, namely 30–650 kHz here. Observations are binned in a 48 × 50 grid, with 0.5 hr wide LT bins and 3.2° wide latitude bins. Data are shown such that observations made in the ecliptic plane at midnight are in the center of the plot. Note that these distributions do not show the complete distribution of Wind viewing positions, but only those in which AKR is present.

The LT distribution in Figure 1b shows increases around noon (12:00 hr), midnight (00:00 hr), dawn (06:00 hr) and dusk (18:00 hr). This is due primarily to the varying orbital trajectory of Wind for the 10 years, and represent the excursions to Lagrangian points as mentioned. As the marginal distributions in Figure 1 show only AKR observations from Wind, it does not show the complete range of viewing positions, namely those from which AKR was not observed due to source inactivity or the distance of the spacecraft from the beamed emission. The relative fraction of measurements for which AKR is observed is much lower on the dayside than on the nightside. The strongest AKR sources are known to be localized to the nightside magnetosphere; this is reflected in Figure 1a, which shows a significant decrease in observed AKR power between ∼06:00 and 18:00 hr LT. There is a sharp decrease at all latitudes in the median AKR power close to 06:00 hr LT which is likely due to AKR sources preferentially found toward dusk, in the premidnight LT sector. If AKR sources activate more readily in a wider range of premidnight compared to postmidnight LTs, the decrease in AKR power will be smoother in this region.

The latitudinal distribution of the Wind AKR observations, shown in Figure 1c, gives the latitude of the spacecraft in solar magnetic coordinates (*λ*_SM_, with positive values aligned with the magnetic pole in the Northern hemisphere), and shows the general bias toward measurements made in the Northern magnetic hemisphere. The majority of observations are made between −40° and 40°, while Wind infrequently reached higher latitudes to a maximum magnitude of 80°. The appearance of the average data at higher latitudes is due to slow, sparse passes of the spacecraft through these regions. The general trend in the AKR power shows an average increase when viewed from higher latitudes in either hemisphere, with decreased power and dependency on latitude in dayside LTs; agreeing with previous observations (Lamy et al., 2010; Waters, Jackman et al., 2021). It should be noted however, that the majority of these observations are made at relatively low latitudes when compared with other studies, that may constrain the viewing of lower latitude or higher altitude AKR sources given recent models of tangent plane beaming (Mutel et al., 2008; Schreiber et al., 2017).

Figures 1d and 1e show the median AKR integrated power again binned in LT, after Fogg et al. (2022), extended to cover the relevant interval 1995–2004. This corroborates their results, with a similar decrease in power seen as Wind observes from dayside LT, out of view of the primary emission from the nightside sources. For this study, where we focus on comparison of AKR bursts with substorm lists, we focus on intervals where Wind was observing from local times between 18:00 and 06:00 hrs LT as these represent the best viewing of the AKR sources.

The data in Figure 1 is derived from 3 min resolution flux density data from Wind, processed with a calibration specific to AKR observations and an automatic selection of data based on the change in intensity across the Wind spin period (Waters, Jackman et al., 2021). Measurements are given in 32 frequency channels between 20 and 1,040 kHz. Note that the 52 kHz channel is often selected but can contain emission not associated with AKR; we replace these flux densities with interpolated values of neighboring channels. This selection allows us to explore the AKR intensity on a statistical basis, given the breadth of Wind data available and simple applicability of the selection algorithm, as well as the coincidence of low‐frequency extensions with other magnetospheric phenomena. This can be done with the flux densities themselves, but also by integrating the power over particular spectral ranges to further characterize the AKR. Fogg et al. (2022) has recently refined the AKR selection, output by Waters, Jackman et al. (2021), to formulate a list of discrete AKR bursts. This process includes steps based on a priori knowledge of the AKR morphology, as seen in dynamic spectrograms, namely that low frequency emission (below 100 kHz) is generally accompanied by AKR at higher frequencies. Morioka et al. (2007) describe the lower frequency, higher altitude AKR sources as existing between 6,000 and 12000 km. The lower of these altitudes corresponds to an upper bound of ∼200 kHz for the lower frequency AKR range. Here, a conservative estimate of 100 kHz is chosen to constrain the behavior of the highest altitude sources. As well as start and end times of clusters of observed AKR emission, or bursts, the output of this processing also parameterizes each burst for spectral information, namely its upper and lower frequency bounds.

**Figure 2:**
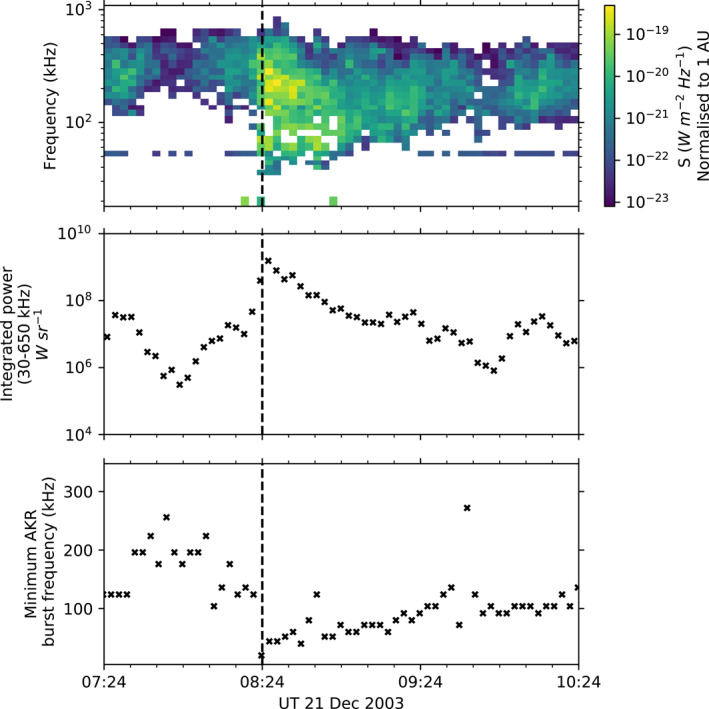
Auroral kilometric radiation (AKR) response during a substorm onset at 08:24 UT on 21 December 2003, as defined by the Substorm Phases from Indices of the Electrojets algorithm with 90% expansion percentile thresholds—(see Section 2.2). The top panel shows the frequency‐time flux density dynamic spectrogram from Wind/WAVES, following the selection of AKR outlined in (Waters, Jackman et al., 2021), for a 3 hr period about onset, which is indicated by the black dashed line. The middle panel shows the corresponding observed radio power, here integrated between 30 and 650 kHz. The bottom panel shows the minimum frequency bound of the AKR burst determined by Fogg et al. (2022).

Figure 2 shows an example of the Wind/WAVES data used in this study; a substorm onset from the Substorm Phases from Indices of the Electrojets (SOPHIE) algorithm is shown (see Section 2.2), with radio data from 60 min before onset to 120 min after onset. The top and middle panels show AKR‐calibrated flux densities and emitted power per unit solid angle respectively, from Waters, Jackman et al. (2021), while the bottom panel shows the minimum frequency bound of the burst associated with the example onset, from (Fogg et al., 2022) The frequency‐time flux density dynamic spectrogram in the top panel of Figure 2 shows AKR emission predominantly between ∼200–500 kHz before onset. Intensifications of at least two orders of magnitude are then seen at most frequencies recorded between this range, while channels sampled below 200 kHz activate as the AKR extends to lower frequencies. Note that the AKR flux densities used here are normalized to 1 AU to account for the various distances at which the observations were made (Waters, Jackman et al., 2021). While the spectral information is lost, the middle panel of Figure 2 shows the radio power integrated between 30 and 650 kHz, which characterizes the AKR response temporally and provides an informative metric over which to compile substorm events. The bottom panel of Figure 2 shows the minimum observed frequency of the AKR burst of Fogg et al. (2022) associated with this substorm, used as a proxy for the average upper altitude bound of the AKR source region.

### 2.2 Substorm Lists

As mentioned in the Introduction (Section 1) substorms have signatures throughout the magnetosphere‐ionosphere system, such as dipolarisation and bursty bulk flows in the magnetotail and strengthening of the westward electrojet in the high latitude ionosphere. They have been characterized by a number of these observational phenomena, initially by visual examination (e.g., the extensive all‐sky camera observations historically used by Akasofu (1964) to describe the main auroral evolution of the substorm) and later with processing of extensive datasets made available by spacecraft observations or large networks of ground magnetometers. These efforts have created a variety of lists of substorm onsets, as defined by these various signatures. Some of these have been retrospectively applied to long‐standing observational datasets, and as such have created lists that span a comparable time range to that of the Wind observations.

In this paper, we consider lists of onsets derived from a single observational proxy. One such observational proxy is derived from the global SuperMAG network of ground magnetometers which forms the SML index (Newell & Gjerloev, 2011), an analogue to the historic AL index (Davis & Sugiura, 1966), which measures the strength of the Westward electrojet. The SOPHIE (Forsyth et al., 2015) algorithm analyses the SML index to select times of significant decreases of the index relative to the considered timespan. This algorithm also uses a free statistical parameter, as only events with decreasing rates of change in SML greater than that given by the expansion percentile threshold (EPT), or a given quantile over the included SML data (Forsyth et al., 2015). In this way, higher EPT values generate a list of substorms with a larger response in the Westward electrojet. Forsyth et al. (2015) published three event lists, with EPT values of 50%, 75%, and 90%. By including a priori knowledge of the observed structure of a substorm and the average duration, further steps are performed to produce a list of the start times of substorm phases, namely the growth, expansion and recovery phases. The published lists each cover the period from 1995 to 2014. Some expansion phases follow recovery phases in the SOPHIE output; these are attributed to intensifications of the substorm as opposed to an initial onset, and are removed from our analysis, where we instead focus only on substorms which have growth, expansion onset and recovery phases in order. Flagged expansion phase onsets, where changes in the SML are similar to changes in SMU and thus may be instead attributed to steady magnetospheric convection, are also removed.

The list by Borovsky and Yakymenko (2017) uses measurements of the specific entropy of the electrons in the nightside dipolar region, made by the Synchronous Orbital Particle Analyser instruments onboard the LANL spacecraft in geosynchronous orbit, to determine whether an injected population due to substorm onset is present. This list is hereafter referred to as the LANL list. A specific entropy of the electron population attributed to the substorm injection is calculated for each of the spacecraft, with a 30 min resolution. Measurements from all the spacecraft are compiled, and the occurrence of a substorm is determined when the minimum specific entropy across all spacecraft decreases by a fixed threshold for recurrent timesteps. As determined by the identification scheme, the minimum time between substorm injections is 60 min. As the measurements are derived from multiple geosynchronous spacecraft that are not necessarily near local midnight, the onset times are subject to a 0–30 min uncertainty due to the time taken for the substorm‐injected population to drift to the position of the spacecraft. The published list covers the period from 1989 to 2007.

McPherron and Chu (2018) published a list that uses ground magnetometers at mid‐latitudes (|*λ*| < 50°) to determine substorm onset, using a typical signature in both the Northward and Eastward components of the magnetic field to derive the mid‐latitude positive bay (MPB) index. McPherron and Chu (2018) use a statistical threshold to define a potential pulse due to substorm, prior to further processing to eliminate short or weak events.

**Figure 3:**
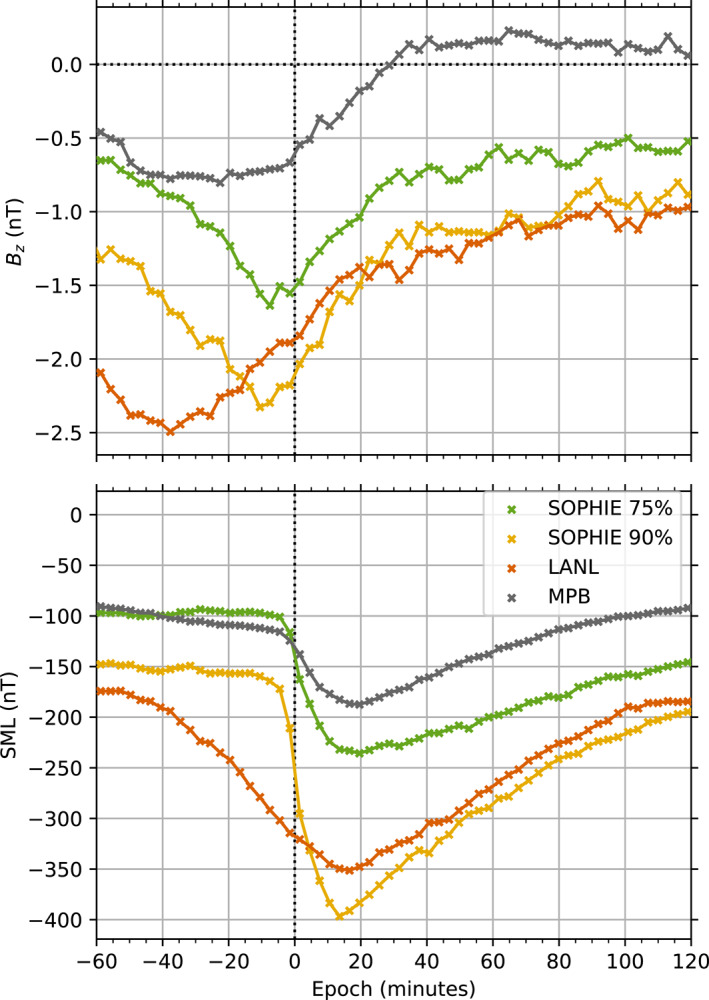
Superposed epoch analysis of (a) solar wind data from OMNIWeb (Papitashvili & King, 2020), showing the median *B*_*Z*_ (z component of the interplanetary magnetic field in geocentric‐solar‐magnetic coordinates) and (b) median SuperMAG (Gjerloev, 2012) SML for a 3 hr window about the time identified as substorm onset by various event lists. The legend denotes the median values derived from the respective event lists. The first two refer to the list derived by Forsyth et al. (2015) which relies on the SuperMAG network of ground magnetometers. Accompanying percentages represent the expansion percentile threshold value used in their algorithm. For the two Substorm Phases from Indices of the Electrojets (SOPHIE) event lists, only the substorm expansion phase onsets are used instead of substorm intensifications (initial instead of multiple successive onsets). The LANL list is that derived by Borovsky and Yakymenko (2017) and uses observations of energetic electron particle injections from the LANL satellites at geosynchronous orbit. The mid‐latitude positive bay (MPB) list is that derived by McPherron and Chu (2018) and uses the MPB index, also derived by ground magnetometers.

Each of the lists used in this study are represented in Figure 3, where each list has been used to perform a superposed epoch analysis of the southward component of the IMF from OMNI, as well as the SML index derived from the SuperMAG network of gound magnetometers, both at 1 min resolution. The median of the respective parameters is computed across 3 min wide bins. Substorm expansion phase onset typically follows a significant period of southward IMF (*B*_*Z*_ < 0), as magnetic flux is loaded into the nightside magnetosphere via dayside reconnection and convection across the polar cap. This is seen prior to onset for each of the included lists, which see southward IMF for an hour prior to onset; SOPHIE lists are displayed with their EPT values in percentages.

The average SML profile from the SOPHIE 75% and MPB lists are comparable in magnitude, with minimum deviations in SML of between −250 and −150 nT. The same is true for the SOPHIE 90% and LANL lists with minimum deviations between −400 and −300 nT. As such, the selection criteria for these latter lists tend to favor larger substorm events. The SOPHIE lists show the effect of using the rate of change in SML as a threshold for event selection, as SML begins to decrease sharply before the epoch. While the SML response from the LANL event list begins to decrease more than 40 min before onset, falling gradually compared to the other lists, this is due to the coarse resolution of the event list as previously discussed.

The median reponse of the IMF *B*_*Z*_ shows the comparative magnitude of the events that are retained by the respective event selection; those with more negative *B*_*Z*_ prior to onset are assumed to produce a greater disturbance within the magnetosphere as this allows for longer periods of ideal IMF conditions to provide magnetic flux to the magnetotail via dayside reconnection. Comparing the median *B*_*Z*_ from the SOPHIE 90% list with that from the LANL list, for example, which have minimum *B*_*Z*_ of between −2.5 and −2.0 nT, suggests that these lists contain larger events. Given that the LANL event list is based on particle injections at geosynchronous orbit, for an event to be retained it requires a substorm of a magnitude that will allow the Earthward‐traveling electron population to reach a distance of at least six *R*_*E*_. It can be assumed that not all substorms will be of the energy to meet this criteria, and so the comparison between the LANL and the SOPHIE 90% event lists is warranted given the 90% quantile threshold applied to SML deflections in the SOPHIE algorithm. Other lists show a more pronounced minimum, with the 75% and 90% SOPHIE lists having similar profiles.

Figure 3 also shows the influence of using the different observational proxies to define onset and encapsulates the various temporal uncertainties inherent in each data set. This is important when interpreting the results of similar analyses performed on the AKR power and other features. Due to the various types of observation and methods of determining substorm onset used here, each superposed epoch analysis is performed over a different number of substorm onsets. AKR has been observed to have a transient spectral response at low frequencies at substorm onset, and correlates with the historic AE index (Morioka et al., 2007, 2010; Voots et al., 1977). With the breadth of AKR data now available from Wind, we explore the extent to which AKR can be used as a similar metric for the onset of substorms, and how the AKR emission relates to the substorm timeline. For Section 3 we assess the AKR power with respect to each of the aforementioned lists.

## 3 Substorm Timeline

Intensifications of AKR are known to coincide with auroral brightenings; it is expected that the average apparent power of the AKR will increase around substorm onset as the auroral oval expands and becomes brighter, signifying the presence of a substorm‐injected electron population which subsequently leads to the generation of AKR. Integrating the AKR power over a particular spectral range gives a proxy of the extent of the source regions along a field line; an increase in power integrated over a given observation frequency range implies the ignition of AKR source regions within an altitude range given by the corresponding electron gyrofrequencies.

With an appropriate list of substorm onsets, such that Wind is an appropriate viewing position, a superposed epoch analysis can be performed on the AKR power. In this way, the average variations in the AKR power with respect to the substorm timeline can be deduced, removing any variations that could be present for single events and not representative of the typical AKR response. For each of the onset lists described in Section 2, we select the substorm onsets in the appropriate period (1995–2004). As mentioned in Section 2, the SOPHIE lists are then reduced to include only expansion phase onsets that follow growth phases (i.e., removing onsets that represent substorm intensifications), as defined by the SOPHIE algorithm. After selecting events to correspond with the observation period of Wind, we further subset the event lists to include only those events which occur when Wind is found in particular LT ranges. The nightside is split into 3‐hr‐wide LT sectors from 18:00 to 06:00, and superposed epoch analyses are conducted for observations from these sectors. For each of the substorm onset times in their respective lists the AKR power across the epoch window is binned in 3‐min wide bins before the median is taken over all of the events. Data where no AKR observations are recorded are excluded from the analysis. Here, the epoch window is taken to be 3 hr (−60 to +120 min about onset). Given that the outputs of both the initial AKR selection and the refined AKR burst selection may contain empty observations, each set of 3 min bins may not be filled for all events from a particular list. Thus, for a given number of substorm onsets, a variable fraction of these contribute to the overall average.

Table 1 shows the number of resulting onsets for each LT sector that are used in the following analysis. The table again reflects the sensitivity of the substorm onset event lists, with the MPB list giving the most events while the LANL and SOPHIE 90% lists, which record stronger substorms, contain the least. It is important to note that the LT selection refers to the observer (Wind) and not the AKR sources themselves. The beaming of AKR and the nature of the remote Wind observations are such that the emission from an AKR source may be observed by Wind when it is at a position up to ∼2 hr away in LT, based on previous observations of AKR and equivalent emission at Saturn (Kimura et al., 2013; Lamy et al., 2008; Schreiber et al., 2017). However, given that the AKR response here is averaged over a significant number of events, and following comparison with results of a superposed epoch analysis using events from a wide LT range centered on midnight (20:00–04:00 hrs), it is likely that the response is attributable to the most intense AKR sources at least close to the corresponding 3 hr wide sectors mentioned above.

**Table 1:** Total Number of Substorm Onsets From Each Event List and for Each LT Range Used in the Superposed Epoch Analyses

Substorm list				
	SOPHIE 75% initial onset^a^	SOPHIE 90% initial onsets^a^	LANL^b^	MPB^c^
Wind LT range (hrs)				
18:00–21:00	470	295	283	1,038
21:00–00:00	471	409	378	1,008
00:00–03:00	647	491	438	1,405
03:00–06:00	974	723	618	2,127
20:00–04:00	1,428	1,114	1,027	3,011

*Note*. LT, local time; MPB, mid‐latitude positive bay; SOPHIE, substorm onsets and phases from indices of the electrojets.

^a^ Forsyth et al. (2015).

^b^ Borovsky and Yakymenko (2017).

^c^ McPherron and Chu (2018).

**Figure 4:**
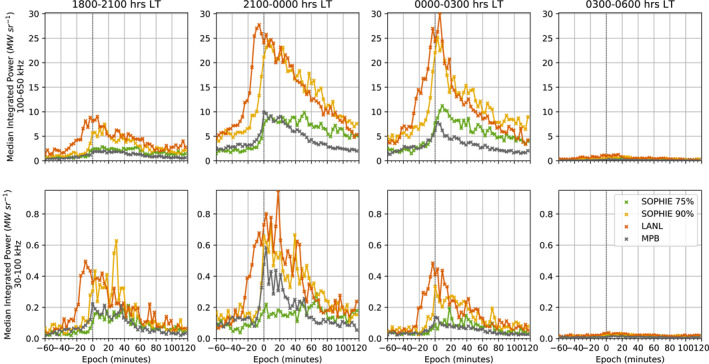
Superposed epoch analyses of the median auroral kilometric radiation (AKR) power about substorm expansion phase onset. The AKR power is given in units of MWsr^−1^ and binned at 3 min resolution, and is shown for a 3 hr window, offset from the onset by 30 min. The AKR power is integrated in two frequency ranges, 100–650 kHz and 30–100 kHz, characterizing what is referred to here as higher frequency (HF) and lower frequency(LF) AKR. The top row of the figure shows the HF AKR response, while the bottom row shows the LF AKR response. Each column shows the AKR response for epochs based on the observation local time (LT) (of Wind), representing 3‐hr‐wide LT sectors covering the nightside from 18:00–06:00. Each line shows the AKR power for a different event list of onsets, denoted in the legend and corresponding to the same event lists as in Figure 3.

Figure 4 shows the results of superposed epoch analyses for each event list and LT sector. Each column of the figure shows results from a different LT range, displayed at the top of the plot. The top row of the figure pertains to the HF AKR response, showing the median AKR power integrated over the frequency range 100–650 kHz, while the bottom row presents the median AKR power integrated between 30 and 100 kHz and thus the LF, higher altitude AKR response. The median AKR power in both the HF and LF frequency ranges show an increase close to onset, although the largest increases are seen in the LT ranges 18:00–21:00, 21:00–00:00, and 00:00–03:00 hrs. The average response observed from 03:00 to 06:00 is barely apparent on a comparable scale; the LF peaks for the 03:00–06:00 LT range reach no more than 10% and 5% of the LF peaks for the 00:00–03:00 and 21:00–00:00 LT sectors, respectively, when comparing the results from the SOPHIE 90% event list. The HF peak of the 03:00–06:00 LT sector reaches no more than 5% of the HF peaks for 21:00–00:00 and 00:00–03:00 LT sectors.

The comparative magnitude of events selected by each list is seen in the AKR response, with the median power for the LANL and SOPHIE 90% lists greatly exceeding that for the SOPHIE 75% and MPB lists, which each have a similar response in magnitude. In the HF, each list sees a gradual increase in the AKR power from 20 to 0 min before the epoch, with an increasingly steep rise to a clear peak in the 20 min after the epoch. For the LF, each list also sees an increase in the AKR power from 20 to 0 min before the epoch, but the peak is seen up to an hour after the epoch. The profiles are noisier in the LF, however, which could be due to the inclusion of less AKR observations at low frequencies and subsequent influence of powerful bursts for a given observation. Particularly prominent peaks appear in the LT ranges 18:00–21:00 hrs for the SOPHIE 90% list, and 21:00–00:00 hrs for the SOPHIE 90% and LANL lists, at ∼20–40 min after onset. This could be indicative of further substorm intensifications occurring. Although changes in the average AKR power profile gained using events from the LANL list tend to precede those from other lists, this is assumed to be due to the aforementioned coarse resolution of the selection algorithm used. While there is a clear gradual rise in AKR power in the 20 min preceding onset, the beginning of the steep increase to the peak is clearly seen between ∼ −20 and −5 min, which could indicate the ignition of more powerful AKR source regions prior to other observable signatures of substorm onset. The idiosyncracies of the lists may have a greater influence than a true AKR response, however. For SOPHIE lists, the AKR response here could reflect the median SML response in Figure 3 which begins to decrease prior to the epoch. The coarse resolution of the LANL event list means the exact time of an onset‐associated response in the corresponding AKR observation may be lost. It is also important to note that the minimum resolution for all frequency channels from the Waters, Cecconi et al. (2021) data set is 3 min, thus the analysis of the AKR coupling timeline during substorm onset is limited by this resolution. While it is clear from Figure 4, then, that the AKR response begins to increase before the identified substorm onset, more work is needed to properly determine the prevalence of an AKR signature as a precursor to substorm onset.

**Figure 5:**
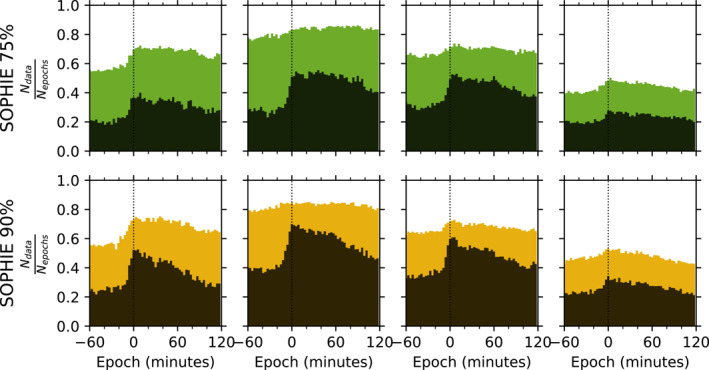
Occurrence of auroral kilometric radiation (AKR) observations in each 3 min bin relative to epoch used in the analysis, for both Substorm Phases from Indices of the Electrojets (SOPHIE) 75% (top row) and SOPHIE 90% (bottom row) event lists. The columns represent local time ranges of analysis in the same way as Figure 4. The colored distribution for each panel represents the higher frequency AKR observations, while the black distribution represents the lower frequency AKR observations.

Figure 5 shows the number of AKR power data included in each 3 min epoch bin, compiled over all events and as a fraction of the total number of onsets in each LT range (as shown in Table 1), for both HF and LF AKR power. Each column of Figure 5 corresponds to a LT range in the same way as Figure 4. The top row of Figure 5 shows the distributions for the SOPHIE 75% list, while the bottom row shows those for the SOPHIE 90% list. Each panel shows the HF power counts in color and the LF power counts in black. Each event list shows a greater increase in the occurrence of LF AKR power at onset than for HF AKR power, in all LT ranges except for 03:00–06:00 where the increase is similar. This difference is most notable for both lists in the 21:00–00:00 range, where HF AKR is persistent throughout the epoch while LF AKR is recorded ∼2 times as often at the epoch than LF AKR at prior times. This is less clear in LT ranges 18:00–21:00 and 00:00–03:00, and could be due to the expansion of the auroral oval to wider longitudes from the typical premidnight brightening location (Milan et al., 2009), thus igniting both low and high altitude AKR sources at wider LT. As well as the greater increase in occurrence of LF than HF AKR power at the epoch, the occurrence is consistently higher for the SOPHIE 90% list, indicating that substorms with larger deviations in SML have a greater likelihood of igniting higher altitude AKR sources, on average.

Comparing the AKR response for the SOPHIE 75% and 90% event lists, for both frequency ranges across the epoch, we see that the power decreases more gradually after the peak at onset for the SOPHIE 75% (weaker substorms) compared to the SOPHIE 90% (stronger substorms) lists. For the average LF AKR power from the SOPHIE 75% list, considering the 21:00–00:00 LT range, this continues to increase past the epoch. This could be due to the fact that intensifications (expansion phase onsets following recovery phases) are removed from the analyses, but we have not discriminated substorms that are followed by an intensification. In these cases, the intensification that follows later in the epoch will have associated auroral dynamics, and so also AKR dynamics. Considering the SOPHIE 75% and 90% event lists are derived from a quantile threshold of the rate of change of SML (the EPT value), it follows that the former list will include more events in total than the latter as events with smaller magnetic fluctuations are retained in the event selection. If those events are also the initial expansion phase onset before multiple intensifications, which may be more likely for a smaller EPT value, then their combined, average effect could produce this.

Table 2 shows the result of aggregating statistics of the HF and LF AKR power for each individual event. For each subset of events by LT range, for the SOPHIE 75% and SOPHIE 90% lists, we take the 90th percentile of the AKR power for each event. These values, which represent the extremes of the AKR power reached during the epoch window, are then averaged using the median, with associated uncertainties given by the relative median absolute deviation (MAD). For each LT and power range, the ratios of the corresponding SOPHIE 90% with the SOPHIE 75% values are taken. In this way, the relative increase in AKR power for stronger substorms in the 3 hr epoch window used here can be characterized for both LF and HF frequency ranges. For all LT and frequency ranges, the average extreme power for events increases for the SOPHIE 90% list over the SOPHIE 75%; this is expected as the differing sensitivities of the event lists (as seen in Figure 3) and the results of Figure 4 indicate a greater AKR power for larger substorms. Within the uncertainties given in Table 2, derived from appropriate error propagation of the corresponding MAD value, the ratio of average extreme power values for HF AKR is lower than LF AKR for all LT ranges except 03:00–06:00. It is unsurprising that this LT range differs from the others, given the weakest response in AKR power was seen here. The discrepancy is most notable for the premidnight LT sector at 21:00–00:00, with the average extreme AKR power in the LF 3.6 ± 0.2 times greater for SOPHIE 90% (stronger) onsets than SOPHIE 75% (weaker) onsets, compared to 2.7 ± 0.1 times greater in the HF range. This corroborates previous studies of the statistical magnetic local time of substorm onset as well as AKR source locations (Milan et al., 2009; Schreiber et al., 2017). The results of Table 2 indicate that the ignition of higher altitude AKR sources is much stronger for larger substorms, and that in turn the activation of the extended auroral acceleration region is higher for these events. It is possible to say that the AKR sources are present at higher altitudes due to the observed emission and increased power. However, the increased intensity of the emission at a certain frequency (and so at a given altitude) could be attributed to a change in the growth rate of the cyclotron maser instability, or the azimuthal extent of the auroral cavity, or both. For this reason, it is difficult to make a direct inference on this without in situ measurements of the acceleration region, especially with a statistical perspective over many events as shown here.

**Table 2:** Ratios and Associated Uncertainties of the Median Power Extremes for Events From SOPHIE 90% and SOPHIE 75%, Shown for HF and LF Frequency Ranges in the Studied LT Ranges

	18:00–21:00	21:00–00:00	00:00–03:00	03:00–06:00
HF	2.2 ± 0.2	2.7 ± 0.1	2.2 ± 0.1	1.7 ± 0.3
LF	2.5 ± 0.2	3.6 ± 0.2	2.9 ± 0.2	1.5 ± 0.2

*Note*. See text for a detailed description of the data aggregation. HF, higher frequency; LF, lower frequency; SOPHIE, substorm onsets and phases from indices of the electrojets.

## 4 Low Frequency AKR Characteristics

The determination of AKR bursts allows us to quantify spectral features such as the bounding frequencies of the bursts and their spectral extent (Fogg et al., 2022). Such parameters can give us further insight into the altitudinal evolution of the auroral acceleration region during the substorm timeline. Namely, the bounding frequencies of the observed bursts allow us to estimate the spatial extremes of the auroral acceleration region in which AKR is generated.

**Figure 6:**
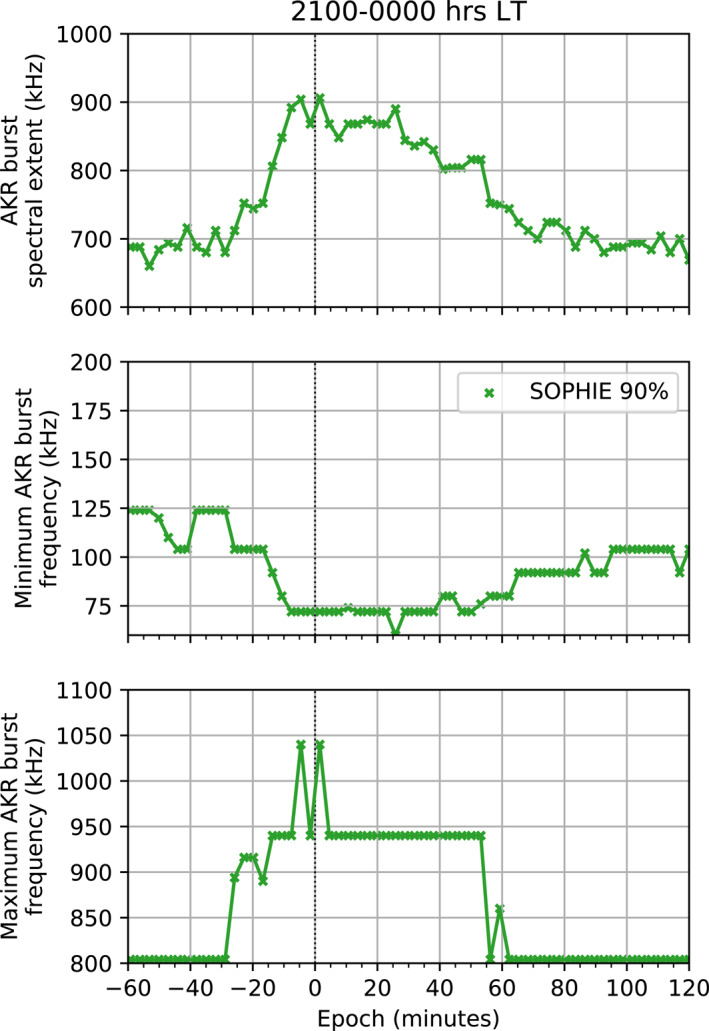
Superposed epoch analyses of auroral kilometric radiation (AKR) burst parameters observed from the 21:00 to 00:00 hr local time (LT) sector. Median burst parameters are shown across the epoch using events from the Substorm Phases from Indices of the Electrojets (SOPHIE) 90% list for the period 1995–2004. The top panel shows the median spectral extent of AKR bursts, while the middle and bottom panels show the median minimum and maximum bounding frequencies of AKR bursts.

Figure 6 shows the evolution of burst parameters that result from a superposed epoch analysis that uses the SOPHIE 90% event list, further subset as previously mentioned. The superposed epoch analysis is similarly conducted over a 3 hr window, with AKR observations binned at 3 min resolution. The top panel of Figure 6 shows the median spectral extent of AKR bursts, or the difference between the maximum and minimum frequency channels in which an AKR burst is observed. This provides information on the vertical extent of the acceleration region, given that the cyclotron‐maser‐instability that generates AKR produces emission at frequencies inversely proportional to the source altitude. For more context to this, and to allow us to quantify the exact altitude of the extremes of the acceleration region, the middle and bottom panel show the median minimum and maximum bounding frequencies of AKR bursts throughout epoch. This allows us to explore how the radio sources grow/move in response to substorm‐associated excitation: for example, we can see whether the low‐frequency component ignition occurs before, simultaneous, or after substorm onset (as defined by complementary datasets in the SOPHIE list). This timing is critical for quantifying the magnetosphere‐ionosphere coupling timescale.

The median spectral extent of AKR burst begins to increase from approximately 700 kHz around 20 min before onset, approximately coinciding with the increase of AKR power in both frequency bands. The spectral extent peaks at ∼900 kHz just after onset for this LT sector. This maximum extent is transient, remaining for 3 min before gradually decreasing to 700 kHz again 80 min after substorm onset. While there is a secondary increase of the spectral extent between 40 and 60 min after onset, this is likely due to the much larger spacing between HF channels and the change in median maximum bounding frequency for this time, as seen in the bottom panel.

The middle panel of Figure 6 shows the clear decrease in frequency of AKR bursts during substorm onset. Around 20 min before onset, the minimum frequency is measured at approximately 100 kHz. This falls to 75 kHz at onset, which is generally consistent following onset. As found previously, but here shown over a statistical basis with many events, this corresponds to an extension of the AKR to low frequencies at substorm onset. However, these results show a persistent minimum frequency which indicates a more sustained increase in altitude of the acceleration region.

Spectral AKR burst parameters are derived from the discrete frequency channels of Wind observations. This limits the accuracy of the estimation of the height of the source region, particularly at lower altitudes (higher frequencies, here greater than ∼200 kHz) where observation channels are logarithmically spaced. At higher altitudes however, corresponding to lower frequency channels between 60 and 148 kHz, the spacing between channels is between 8 and 20 kHz, with an average spacing of approximately 12 kHz between the eight frequency channels in this range. This corresponds to an altitude range of ∼1,000 km, assuming the source location is given by the electron gyrofrequency equivalent to the emission frequency and lies on a magnetic field line with an invariant latitude of 70° (as included in and estimated from Figure 3 of (Morioka et al., 2007)). We note that the invariant latitude used to calculate the emission altitudes is higher than the ∼65° typical of substorm onset.

The bottom panel of Figure 6 shows the median maximum bounding frequency of AKR bursts during the substorm timeline. The maximum frequency is mostly consistent in the 30 min before onset, at 800 kHz. As well as for the lowest frequencies however, the highest frequencies of emission also change around 20 min before onset, increasing to measurements at 1,040 kHz 10 min before onset. The maximum frequency reaches a peak after onset at the maximum frequency channel of the Wind observations used here, at 1,040 kHz. Although there are limitations based on the measuring capabilities of the Wind/Waves RAD1 instrument, as previously mentioned, it is clear that emission that is fairly characterized as AKR is present here.

These results show conclusively that the response of the range of emission frequencies of AKR begins to extend around 20 min prior to substorm expansion phase onset as determined by SOPHIE. As well as the results from Section 3, it is clear that the AKR response precedes substorm onset. This highlights the potential usefulness of the average AKR response as an indicator of substorm onset, particularly given that the low‐frequency extensions are apparently exclusive to substorm dynamics. However, more study of the conditions presiding over AKR emission and the occurrence of AKR source dynamics is needed to constrain this understanding.

## 5 Summary

AKR sees enhancements in intensity and changes in frequency, and has been postulated to be associated with other dynamics in the terrestrial magnetosphere such as auroral brightenings and discrete arcs, earthward bulk flows of electrons and strengthenings of high latitude current systems. Previous studies have explored the AKR variability alongside these phenomena, which are also closely associated with substorm dyanmics, but have used AKR observations that cover a few months or studies that include only a few tens to a hundred events. Here we use observations of AKR from Wind, made between 1995 and 2004, that coincide with published lists of substorm events to expand upon previous studies and further examine the average AKR response during the substorm timeline. We integrate the AKR power over two important frequency ranges that best characterize the spectrum, covering higher and lower frequency portions. To infer the evolution of the acceleration region, we also examine the observed spectral extent of the AKR bursts as well as the minimum and maximum bounding frequency. We use a variety of substorm lists, including those output from the SOPHIE algorithm at EPT values of both 75% and 90%, that derived from the MPB index, and the list derived from the geosynchronous LANL satellites and their measurements of electron populations. The SOPHIE and MPB index lists are themselves derived from ground magnetometer observations. As an initial comparison of the substorm lists themselves, we perform a superposed epoch analysis of the *B*_*Z*_ component of the IMF, shown in Figure 3. This demonstrates the sensitivity of each substorm list; those with a larger southward component prior to onset indicate a list containing the largest events. To ensure observations with appropriate viewing of the nightside AKR sources are retained in the analysis of the AKR features, substorm events are subset by the LT of Wind at the time of expansion phase onset. Once subset in this way, both the AKR power and spectral features of AKR bursts are examined in superposed epoch analyses for each of the substorm lists.

Figure 4 shows the results of the superposed epoch analysis of the AKR power for each substorm list, with events subset into four LT sectors, each 3 hr wide, covering the entire nightside from dusk to dawn. Separate analyses are conducted for the frequency range that characterizes the lower frequency AKR component (30–100 kHz) as well as for the HF component (100–650 kHz). These results show that the primary AKR response is centred pre‐midnight, and is mostly confined to the sectors neighboring midnight (LT sectors 21:00–00:00 hr and 00:00–03:00 hr). The sensitivity of the substorm lists to event size is also seen in the response of the AKR power, with a larger magnitude response for the LANL and SOPHIE 90% lists. Figure 4 also shows a response in the AKR power, for all lists, and in both frequency ranges, prior to the onset epoch time. While this suggests that AKR enhancements precedes the other typical signatures of substorm onset shown here, more work is needed to assess the influence of the uncertainty of the event lists. While the timing details can be better constrained, the results here suggest that such analyses of AKR observations may provide important hints on the formation of the Alfvénic double layer. The distribution of AKR power data throughout the epoch for both HF and LF frequency ranges, and both SOPHIE 75% and 90% event lists, is shown in Figure 5. This shows a greater increase in occurrence of LF AKR at onset than HF AKR for LT from 21:00 to 03:00, and that there is a greater likelihood of LF AKR for the stronger events of SOPHIE 90%. The discrepancy between HF and LF occurrence is greatest for pre‐midnight observations, corresponding to the typical substorm location. To highlight the differing AKR response between substorms of different strengths, we compare directly the average response from the SOPHIE lists with 75% and 90% EPT values. Table 2 shows the increase in averaged, extreme power values during each event, with LF AKR power values 3.6 ± 0.2 times greater for stronger events from the SOPHIE 90% event list than those of the SOPHIE 75%, while HF AKR has values 2.7 ± 0.1 times greater.

The results of comparing the AKR burst parameters with the SOPHIE 90% event list show the average evolution of the nightside AKR source region, viewed remotely, as it extends vertically. Figure 6 shows that the response of the AKR power during substorm onset is attributable to this vertical extension of the AKR sources, and the auroral acceleration region by proxy. Our work, based on a decade of high fidelity radio data from Wind/WAVES has shown the utility of the AKR as a proxy for magnetospheric dynamics. In particular, we track the increase in radio power and the expansion in frequency of the spectral signature associated with substorm expansion phase onset for 10 years of observations where Wind is suitably located. The timing of the AKR response has been compared between the event lists and show a similar time profile to averages of corresponding indices such as SML, while the greater increase in AKR power for stronger events is likely due to a greater occurrence of LF AKR. While important to acknowledge the temporal uncertainties present in the event lists, further study of the time and magnitude of AKR intensification across events of various sizes can show insightful disparities in the auroral acceleration region via AKR. The utility here suggests that AKR integrated power can be employed more widely by the magnetopsheric/ionospheric community as another geomagnetic index to track the global impact of variable space weather.

## Data Availability

Both the AKR‐selected (Waters, Cecconi et al. (2021), https://doi.org/10.25935/wxv0-vr90) and AKR burst data (Fogg et al. (2021), https://doi.org/10.25935/hfjx-xx26) from Wind/WAVES used in this study can be accessed online.
